# Intracaval Migration of Ureteral Stent

**DOI:** 10.5334/jbr-btr.842

**Published:** 2015-09-15

**Authors:** M. Hajji, M. S. Bennani, S. Bekkali, L. Jroundi

**Affiliations:** 1 Department of Radiology and medical imaging, HIMMV, Rabat, Morocco; 2 Department of Urology, HMIMV, Rabat, Morocco; 3 INO Institut National d’Oncologie, Rabat, Morroco

**Keywords:** Stents, prosthesis

## Abstract

Ureteral stents have proven to be an invaluable tool for endourologists. Morbidity is minimal, but complications do exist. Up to 3 months complications are not frequent, but longer indwelling times are associated with increasing frequency of incrustation, infections, secondary stone formation, obstruction of the stented tract and migration.

We report a rare case of a 33 year old pregnant patient with migration of an ureteral endoprosthesis. The patient received a right ureteral stent at 12 weeks for acute obstructive pyelonephritis. When her urologist tried to remove the ureteral stent post delivery, the stent was not found in the bladder. Ureteroscopy was performed but no ureteral stent was found. The patient showed a moderate improvement of the pyelonephritis, but complained about insidious palpitations.

A CT scan was performed and showed the presence of the ureteral stent extending from the inferior vena cava up to the right atrium. Endovascular retrieval was performed through a puncture of the common femoral vein, using a curved guide that was introduced through the vena cava into the right atrium. Under fluoroscopic control, it was twisted around the stent and pulled out. The outcome was favorable, and no other complications were noted.

The use of double pig-tail ureteral catheters has become routine in urological practice nowadays. The frequency of side effects is usually low, and complications are usually minimal. We report a case of intravenous stent migration in a 33 year old pregnant women through a simultaneous perforation of the right ureter to the right common iliac vein, with extension up to the right atrium.

To our knowledge, only few similar cases were reported. Vascular complications of ureteral stent are rare, and usually present as arterio-ureteral fistulas. Their frequency can be increased by several conditions such as degenerative iliac artery disease [1], previous vascular reconstruction procedures [2], extirpative surgery for pelvic or abdominal malignant disease [3], pelvic irradiation, urolithiasis, urinary diversion procedure [4], extensive ureteral mobilisation and prolonged indwelling ureteral stent [5]. Venous complications due to ureteral stenting are less common than arterial ones.

## Case report

We report the rare case of a 33 years old pregnant woman with an acute gestational obstructive pyelonephritis. A right ureteral stent was placed at the twelfth pregnancy week in a private institution with a good evolution of the symptoms.

**Figure 1:**
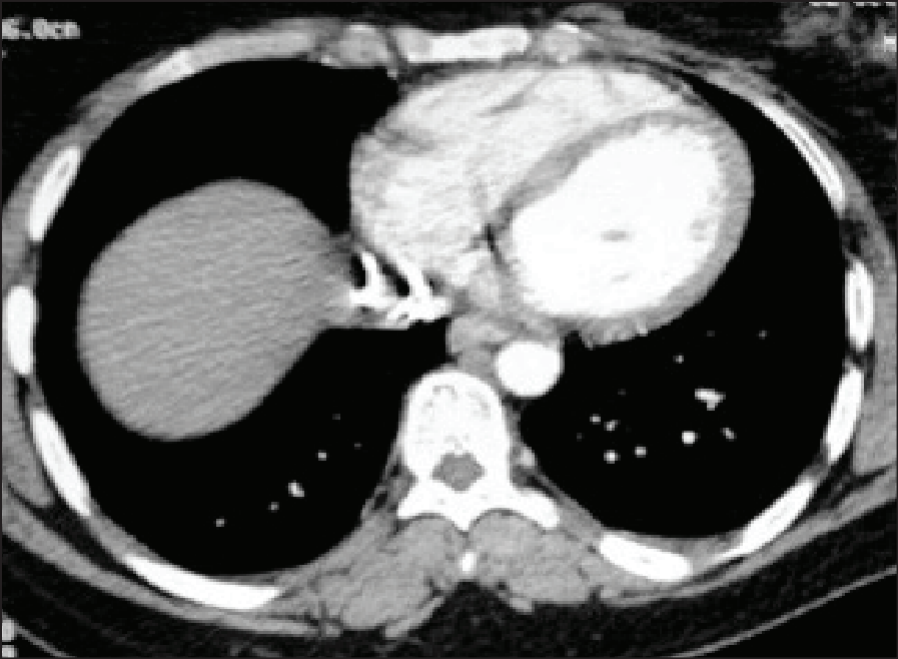
CT showing stent location in vena cava.

**Figure 2:**
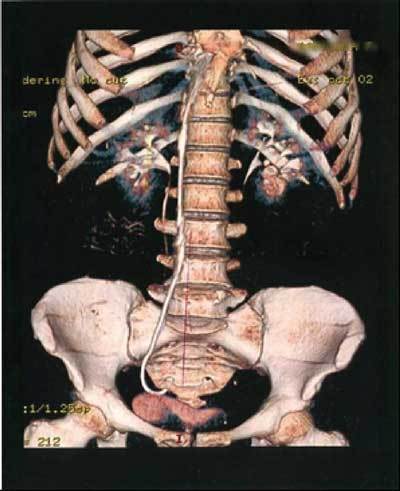
Computed tomography, 3D reconstruction, showing the catheter in the abdominal area.

When time came to remove the stent after the pregnancy, the urologist could not find it nor in the bladder, neither in the all urinary tract at ureteroscopy. A thoraco-abdomino-pelvic computed tomography (CT) was performed and showed that the stent had moved intravascularly. It had migrated through the right iliac vein into the inferior vena cava and right atrium (Figs. 1, 2). The patient was sent to our institution for extraction.

The patient complained about insidious onset of palpitations and moderate right flank pain. She had no macroscopic hematuriprobably due to intracatheter blood clotting.

The patient was then transferred to the vascular surgery department and an endovascular extraction was performed by puncture of the femoral vein. Extraction of the endoprosthesis was made by a curved guide introduced through the vena cava up to the right atrium under fluoroscopic control. The guide was subsequently twisted around the stent and pulled it out. No other complication was noted and the patient was discharged on the following day.

## Discussion

Ureteral double J (pigtail) stenting, either retrograde or antegrade is usual in urological practice as well as in radiological interventional procedures. Modern “double pigtail” ureteral stents are soft and designed to curl at both ends to prevent organ trauma and stent migration [6,7]. These stents are used for a wide variety of conditions. Renal or urocystic migration is commonly seen but intravenous stent migration is exceptional. Michalopoulos et al [8] were the first in reporting a case of intravascular migration of a J-stent placed in the right ureter that migrated during the procedure into the venous blood flow through one of the intercommunicating ovarian veins. In both the previous reported cases, as well as in this one, it is believed that migration was due to ureteral wall fragility, caused by chronic ureteral trauma due to prior urolithiasis and/or ureteral wall inflammation. In our case, the patient did not present any postprocedural clinical or hemodynamical complication. Furthermore. the J stent placement procedure seemed uneventful.

Iliac vessels trauma is responsible of 30% of mortality, mostly from uncontrolled hemorrhage and is a potentially a devastating complication if not immediately diagnosed. Retroperitoneal hemorrhage was preoperatively excluded in our patient by contrast enhanced abdomino-pelvic computed tomography. Furthermore, Bergqvist et al reported a great difference of mortality rates that are low for patients undergoing surgery with a preoperative diagnosis of an arterio—ureteral fistula (0% mortality rate for 42 patients) compared to patients whose diagnosis became clear during surgery (39% mortality rate for 18 patients). Because venous-ureteral fistulas reported in the literature are rare there is no data of mortality rates of this condition. We can assume that establishing preoperative diagnosis is crucial in order to offer the best treatment. In our case the CT scan permitted to establish the preoperative diagnosis.

## Conclusion

Ionic radiation has to be limited during pregnancy in endourological procedures: therefore one must always be aware of the possibility of ureteral stent migration during blind placement. Ultrasound may therefore may be of great help for the diagnosis and treatment of stent misplacement.

## Competing Interests

The authors declare that they have no competing interests.
